# Tau deletion impairs intracellular β-amyloid-42 clearance and leads to more extracellular plaque deposition in gene transfer models

**DOI:** 10.1186/1750-1326-9-46

**Published:** 2014-11-10

**Authors:** Irina Lonskaya, Michaeline Hebron, Wenqiang Chen, Joel Schachter, Charbel Moussa

**Affiliations:** Department of Neuroscience, Laboratory for Dementia and Parkinsonism, Georgetown University Medical Center, 3970 Reservoir RD, Washington, DC, 20057 USA; Department of Traditional Chinese Medicine, Xuanwu Hospital, Capital Medical University, Beijing, 100053 China; Neuroscience Discovery, Merck Research Laboratories, 770 Sunneytown Pike, West Point, PA 19486 USA

**Keywords:** Tau, Intracellular Aβ1-42, Plaques, Autophagy, Proteasome

## Abstract

**Background:**

Tau is an axonal protein that binds to and regulates microtubule function. Hyper-phosphorylation of Tau reduces its binding to microtubules and it is associated with β-amyloid deposition in Alzheimer’s disease. Paradoxically, Tau reduction may prevent β-amyloid pathology, raising the possibility that Tau mediates intracellular Aβ clearance. The current studies investigated the role of Tau in autophagic and proteasomal intracellular Aβ1-42 clearance and the subsequent effect on plaque deposition.

**Results:**

Tau deletion impaired Aβ clearance via autophagy, but not the proteasome, while introduction of wild type human Tau into Tau^−/−^ mice partially restored autophagic clearance of Aβ1-42, suggesting that exogenous Tau expression can support autophagic Aβ1-42 clearance. Tau deletion impaired autophagic flux and resulted in Aβ1-42 accumulation in pre-lysosomal autophagic vacuoles, affecting Aβ1-42 deposition into the lysosome. This autophagic defect was associated with decreased intracellular Aβ1-42 and increased plaque load in Tau^−/−^ mice, which displayed less cell death. Nilotinib, an Abl tyrosine kinase inhibitor that promotes autophagic clearance mechanisms, reduced Aβ1-42 only when exogenous human Tau was expressed in Tau^−/−^ mice.

**Conclusions:**

These studies demonstrate that Tau deletion affects intracellular Aβ1-42 clearance, leading to extracellular plaque.

**Electronic supplementary material:**

The online version of this article (doi:10.1186/1750-1326-9-46) contains supplementary material, which is available to authorized users.

## Background

Two major pathologies that are linked to Alzheimer’s disease (AD), include extracellular β-amyloid (Aβ) plaques and intracellular neurofibrillary tangles (NFTs) comprised of hyper-phosphorylated Tau (p-Tau) [1]. Although Aβ peptides initially appeared to act upstream of Tau pathology in AD [2, 3], more recent data suggest that Tau mediates Aβ toxicity since reduction of endogenous Tau levels attenuates Aβ-induced neurodegeneration [4, 5]. Tau may be a critical mediator of Aβ toxicity in AD [6, 7]. The level of insoluble p-Tau accumulation positively correlates with neurodegeneration and cognitive decline [8], suggesting that Tau dysfunction underlies dementia [9]. Interestingly, some aged human brains develop plaques with no dementia or major cognitive decline [10, 11], while neocortical and hippocampal Aβ and Tau together are often associated with dementia [12]. However, Tau mutations or modifications are causal to some neurodegenerative diseases without plaques, including fronto-temporal dementia linked to chromosome 17 with Parkinsonism (FTDP-17), progressive supranuclear palsy (PSP), and corticobasal degeneration (CBD), suggesting Tau associated neurodegeneration without Aβ deposition [6, 7]. Taken together these findings suggest that Tau is a critical regulator of Aβ1-42 toxicity through clearance of toxic intracellular Aβ1-42 [13] and modulation of extracellular plaque deposition, thus counteracting the toxic effects of Aβ1-42. The interplay between Aβ and Tau suggests that Tau mediates the development and progression of neurodegeneration or it modulates Aβ clearance and contributes to protection.

To evaluate whether Tau function affects intracellular Aβ clearance and alters extracellular plaque formation, we used lentiviral gene transfer models to focus on intracellular Aβ1-42 clearance in wild type and Tau^−/−^ mice. Intracellular Aβ may be cleared via autophagy and/or the proteasome [14, 15]. We previously demonstrated that lentiviral Aβ1-42 expression leads to p-Tau accumulation and inhibition of both the proteasome and autophagy [13, 14, 16, 17], while the Abl tyrosine kinase inhibitor Nilotinib increases autophagic Aβ and p-Tau clearance, leading to decreased plaque levels in AD models [14, 16]. Here we present evidence in primary hippocampal neurons and in mouse brain that Tau expression is critical for autophagic amyloid clearance. Our data suggest that Tau deletion inhibits autophagic flux, resulting in reduction of intracellular Aβ degradation and increased plaque deposition.

## Results and discussion

### Autophagy and the proteasome contribute to p-Tau and Aβ1-42 clearance

**Figure 1:**
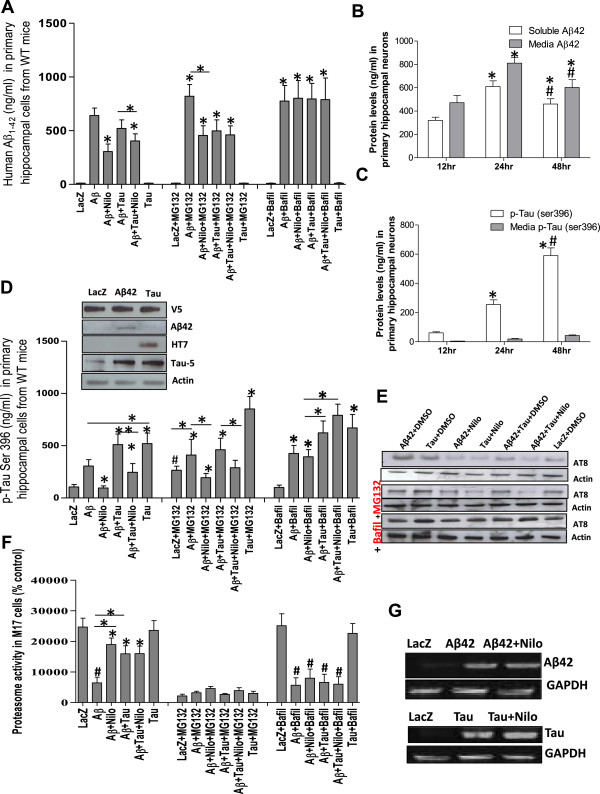
**Inhibition of the proteasome or autophagy partially affects Aβ1-42 and p-Tau clearance.** To dissect out the contribution of the proteasome from autophagy-lysosome in amyloid clearance, primary hippocampal neurons (DIV14) were infected with lentivirus plasmids, and then treated with 1 μl DMSO or autophagy modulators (Nilotinib or Bafilomycin-A1) and/or proteasome inhibitor (MG132). Histograms represent ELISA concentrations of **A)** Aβ1-42 and time course showing the distribution of intracellular and media **B)** Aβ1-42 and **C)** p-Tau Ser 396. **D)** p-Tau Ser 396 in the presence of modulators of autophagy and the proteasome. **Insert**). WB analysis on 4-12% NuPAGE SDS gel showing expression of the lentiviral tag V5, Aβ1-42, human Tau (HT7) and total Tau relative to actin. **E)** WB analysis on 10% NuPAGE SDS gel showing AT8 levels relative to actin. **F)** Histograms represent 20S proteasome activity assay in human M17 neuroblastoma cells. **G)** RT-PCR showing the effects of Nilotinib on lentiviral gene expression relative to GAPDH. # indicates significantly different to LacZ, Asterisk is significantly different to Aβ1-42 + DMSO or as indicated, bars are mean ± SEM, two-way ANOVA.

We previously demonstrated that impaired autophagic clearance of intracellular Aβ leads to more plaque deposition in parkin deficient mice [13, 14, 16, 17]. To determine the contribution of autophagic and proteasomal Aβ1-42 clearance with and without Tau over-expression, primary neuronal hippocampus cultures were infected after 14 days *in vitro* (DIV) with lentiviral constructs driving the expression of human Aβ1-42 or wild type (WT) human Tau for 24 hrs. We previously showed that Nilotinib promotes autophagic clearance of Aβ1-42 [14, 18]. To selectively enhance autophagic protein clearance, neurons were treated with 10 μM Nilotinib (or 1 μL DMSO) for 24 hrs (all cells were treated with DMSO unless Nilotinib was present). To inhibit autophagy, neurons were treated with 100 nM Bafilomycin-A1, and to block the proteasome neurons were treated with 20 μM MG132 for 6 hrs. As expected, Nilotinib significantly decreased human Aβ1-42 levels (Figure 1A, n = 5, p < 0.04) compared to DMSO (1 μL). Nilotinib also significantly decreased Aβ1-42 levels (Figure 1A, p < 0.05) when Tau was co-expressed with Aβ1-42. No human (or mouse) Aβ1-42 was observed when Tau was expressed alone. MG132 significantly increased Aβ1-42 (n = 5, p < 0.043) compared to DMSO in neurons expressing lentiviral Aβ1-42, indicating that some Aβ1-42 is cleared via the proteasome. The combination of Nilotinib and MG132 significantly reduced Aβ1-42 compared to MG132 alone (p < 0.031), indicating that Aβ1-42 may be cleared via autophagy and/or the proteasome. Nilotinib did not change Aβ1-42 levels in neurons co-expressing Tau and Aβ1-42 in the presence of MG132, but under these conditions (MG132 and Tau) Aβ1-42 was significantly lower than MG132 (p < 0.03). Bafilomycin-A1 significantly increased Aβ1-42 (n = 5, p < 0.045) compared to DMSO. In the presence of Bafilomycin-A1, Nilotinib was unable to lower Aβ1-42 levels, further indicating that Aβ1-42 is partially cleared through autophagy.

We previously reported that lentiviral Aβ1-42 expression leads to elevation of p-Tau in the rat cortex [13, 14]. To determine whether autophagic blockade and/or proteasomal inhibition affect amyloid secretion, we measured Aβ1-42 and/or Tau in cell extracts (STEN buffer) and media. Lentiviral expression of human Aβ1-42 in primary mouse hippocampal neurons led to a significant increase in soluble and secreted (media) Aβ1-42 (Figure 1B, p < 0.001, n = 5) at 24 hrs compared to 12 hrs post-infection. Prolonged expression of lentiviral Aβ1-42 for 48 hrs resulted in lower levels of soluble and media Aβ1-42 compared to 24 hrs, but remained higher than 12 hrs (p < 0.01). The level of Ser 396 p-Tau was increased (Figure 1C, p < 0.001, n = 5) with a concomitant increase in media p-Tau (Figure 1C, p < 0.05, n = 5) when Aβ1-42 was expressed for 24 hrs compared to 12 hrs, indicating that Aβ1-42 expression triggers murine p-Tau. p-Tau levels were further increased (p < 0.0001, n = 5) at 48 hrs, suggesting progressive accumulation of p-Tau in response to Aβ1-42.

Because of the observed effects of Aβ1-42 on p-Tau, we also measured p-Tau in cell extracts via ELISA in parallel with Aβ1-42 as shown in Figure 1A. Nilotinib prevented Aβ1-42-induced p-Tau (Figure 1D, n = 5, p < 0.03) compared to DMSO (1 μL). Lentiviral expression of human WT Tau and Aβ1-42 together (DMSO) increased p-Tau (Figure 1D, n = 5, p < 0.03) compared to Aβ1-42 alone but Nilotinib reversed p-Tau (Figure 1D, p < 0.04) back to the level of Aβ1-42 expression alone.

Proteasome inhibition (MG132) increased p-Tau in LacZ infected cells (Figure 1D, n = 5, p < 0.05) or in the presence of Aβ1-42 (+DMSO). However, Nilotinib prevented p-Tau accumulation even in the presence of MG132 in Aβ1-42 expressing cells (n = 5, p < 0.036). Nilotinib also reduced p-Tau (Figure 1D, n = 5, p < 0.03) in cells co-expressing Tau and Aβ1-42 together, further suggesting that autophagy co-operates with the proteasome to clear p-Tau. MG132 significantly increased p-Tau in Tau expressing cells (p < 0.001).

Bafilomycin-A1 robustly increased p-Tau in Aβ1-42 (+DMSO) infected cells (Figure 1D, n = 5, p < 0.034) and Nilotinib had no effect on p-Tau with Bafilomycin-A1. Lentiviral expression was verified by Western blots (WB) showing equal levels of V5 lentiviral tag (Figure 1D insert, 1^st^ blot) in cells expressing lentiviral human Aβ1-42 (2^nd^ blot) or human Tau (3^rd^ blot) compared to total Tau levels (4^th^ blot) relative to actin (5^th^ blot). The ELISA results of Tau metabolism were confirmed with WB using AT8 antibody (Figure 1E, n = 4). Tau or Aβ significantly (p < 0.05) increased p-Tau compared to LacZ (1^st^ blot) or in the presence of MG132 (3^rd^ blot) or Bafilomycin-A1 (5^th^ blot) relative to actin. Nilotinib reduced p-Tau relative to actin (1^st^ blot, p < 0.05) when Aβ1-42 and Tau were expressed together or separately in the presence or absence of MG132, while Bafilomycin-A1 blocked the effects of Nilotinib on p-Tau reduction.

To further determine the effects of Aβ1-42 and p-Tau on proteasome activity, a chymotrypsin-like assay in M17 neuroblastoma showed that Aβ1-42 (with DMSO) significantly decreased proteasomal function (Figure 1F, n = 6, p < 0.001) compared to LacZ (DMSO), but Nilotinib partially reversed Aβ1-42 effects on proteasome function (p < 0.01) in cells expressing Aβ1-42 alone or together with Tau. Tau did not affect proteasomal function, but Tau and Aβ1-42 together significantly reduced proteasome activity (p < 0.04) compared to Tau or LacZ. MG132 completely inhibited the proteasome. However, Bafilomycin-A1 did not affect proteasomal activity in control (LacZ) or Tau expressing cells, but significantly decreased it in Aβ1-42 expressing cells with and without Tau, further suggesting that lack of Aβ1-42 clearance affects proteasome activity. The effects of Nilotinib on lentiviral human Aβ1-42 and Tau expression were verified by RT-PCR using the same primers that were utilized to clone Aβ1-42 and Tau into the lentivirus as we previously described [13, 15, 19]. Nilotinib did not affect Aβ1-42 (Figure 1G, top blot) and Tau (Figure 1G, bottom blot) RNA levels compared to DMSO relative to GAPDH, suggesting that Nilotinib does not alter lentiviral expression.

### Tau is required for autophagic amyloid clearance

**Figure 2:**
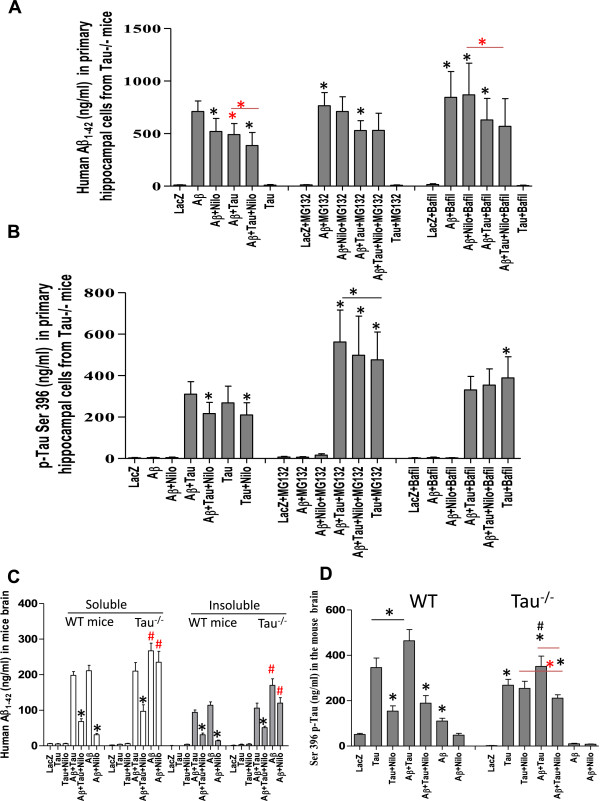
**Tau deletion impairs autophagic clearance.** Primary hippocampal neurons from WT and Tau^−/−^ mice were infected with lentivirus plasmids and then treated with 1 μl DMSO or autophagy modulators (Nilotinib or Bafilomycin-A1) and/or proteasome inhibitor (MG132). Histograms represent ELISA concentrations of soluble and insoluble brain extracts of **A)** human Aβ1-42 and **B)** p-Tau Ser 396. WT (C57BL/6) and Tau^−/−^ mice were injected with 1×10^6^ multiplicity of infection (MOI) of lentiviral human Tau, Aβ1-42, Tau ± Aβ1-42 and adjusted with LacZ. All animals were treated 3 weeks post-injection with daily 10 mg/kg IP injection or 30 μL DMSO once a day for 3 (additional) consecutive weeks. Histograms represent ELISA concentrations of total brain extracts of **C)** human Aβ1-42 and **D)** p-Tau Ser 396. Asterisk is significantly different to Aβ1-42 + DMSO or as indicated, # indicates significantly different to Aβ1-42 + DMSO in WT mice. Bars are mean ± SEM, two-way ANOVA.

To focus on the effects of Tau deletion on intracellular Aβ1-42 clearance, we tested our model in WT and Tau^−/−^ mouse primary hippocampal neurons *in vitro*. Mouse hippocampal neurons were prepared from C57BL/6 (WT) and homozygous Tau^−/−^ mice [20, 21] and infected with the lentiviral clones at DIV14. Nilotinib reduced the levels of Aβ1-42 in Tau^−/−^ neurons (compared to DMSO, p < 0.047) and in cells expressing Aβ1-42 and Tau together (Figure 2A, n = 5, p < 0.032). Interestingly, Aβ1-42 and Tau together (+DMSO) reduced Aβ1-42 levels (p < 0.042) compared to Aβ1-42 alone (+DMSO) and Nilotinib further decreased Aβ1-42 (Figure 2A, p < 0.035), suggesting that Tau expression partially increases Aβ1-42 clearance. MG132 blocked Nilotinib-induced Aβ1-42 clearance in Tau^−/−^ neurons, but Tau expression reduced Aβ1-42 levels (p < 0.037). No Aβ1-42 was detected in Tau expressing Tau^−/−^ primary neurons. Bafilomycin-A1 increased Aβ1-42 levels (p < 0.05) and Nilotinib did not reverse these effects in Tau^−/−^ neurons, but Tau expression reduced Aβ1-42 back to DMSO levels (p < 0.05), further suggesting that Tau expression enhances amyloid clearance. No p-Tau was detected in Aβ1-42 expressing Tau^−/−^ neurons, but Nilotinib significantly (Figure 2B, n = 5, p < 0.05) reduced p-Tau compared to DMSO in Tau alone or with Aβ1-42. MG132 increased p-Tau levels (p < 0.03) even in the presence of Nilotinib when Tau was expressed alone or together with Aβ1-42. However, both MG132 and Bafilomycin-A1 blocked Nilotinib-induced p-Tau decrease (p < 0.01) in the presence of Tau alone or together with Aβ1-42. Together, these data suggest that Tau is required for complete amyloid clearance.

These results were also conducted *in vivo* in WT (C57BL/6) and Tau^−/−^ mice, which are relevant to our experiments because organelle movement was reported to be impaired in these mice [20, 21], which my potentially affect autophagosome movement. Mice were stereotaxically injected into the hippocampus with V5-tagged lentiviral constructs driving human Tau and Aβ1-42 expression and adjusted with LacZ to 1×10^6^ multiplicity of infection (MOI). Animals were treated 3 weeks post-injection with daily 10 mg/kg intraperotineal (I.P) injection of Nilotinib or 30 μL DMSO once a day for 3 (additional) consecutive weeks as we previously reported [14, 16]. To verify equal expression of lentiviral clones, 20 μm thick coronal brain sections were co-stained for human specific Aβ1-42, p-Tau and V5 as shown in Additional file 1: Figure S1. No human (or mouse) Aβ1-42 was detected by ELISA in human Tau expressing WT mice (Figure 2C, n = 4), but Nilotinib reduced Aβ1-42 levels when Aβ1-42 was expressed alone (p < 0.0001) or together with human Tau (p < 0.001). Human Aβ1-42 was significantly higher (p < 0.01) in Aβ1-42 expressing Tau^−/−^ mice compared to Aβ1-42 expressing WT mice (Figure 2C, n = 4). Nilotinib failed to reduce Aβ1-42 levels in Tau^−/−^ mice compared to WT, but introduction of lentiviral human WT Tau with Aβ1-42 into Tau^−/−^ mice significantly reduced Aβ1-42 (p < 0.05) compared to Aβ1-42 alone. These data suggest that exogenous human Tau facilitates autophagic Aβ1-42 clearance. It is important to note that no changes were observed with mouse Aβ1-42 when lentiviral human Tau or Aβ1-42 were expressed in WT or Tau^−/−^ mice (data not shown), and no changes were detected in APP cleaving enzymes [14], indicating no changes in APP processing.

The effects of Nilotinib on p-Tau clearance were also examined. Nilotinib reduced p-Tau (Figure 2D, n = 4, p < 0.01) in Tau over-expressing WT mice and when Tau and Aβ1-42 were expressed together (p < 0.015), which led to more p-Tau than Tau alone (p < 0.02). However, p-Tau was significantly lower (Figure 2D, n = 4, p < 0.037) in human Tau expressing Tau^−/−^ compared to WT mice, and although Aβ1-42 together with Tau significantly increased p-Tau in Tau^−/−^ mice (p < 0.04), p-Tau remained significantly lower (p < 0.05) in Tau^−/−^ compared to WT mice. Nilotinib did not change p-Tau when human Tau was expressed alone in Tau^−/−^ mice, but it significantly reduced it (p < 0.03) when Aβ1-42 and Tau were co-expressed.

### Tau deletion impairs autophagic flux in mouse brain

**Figure 3:**
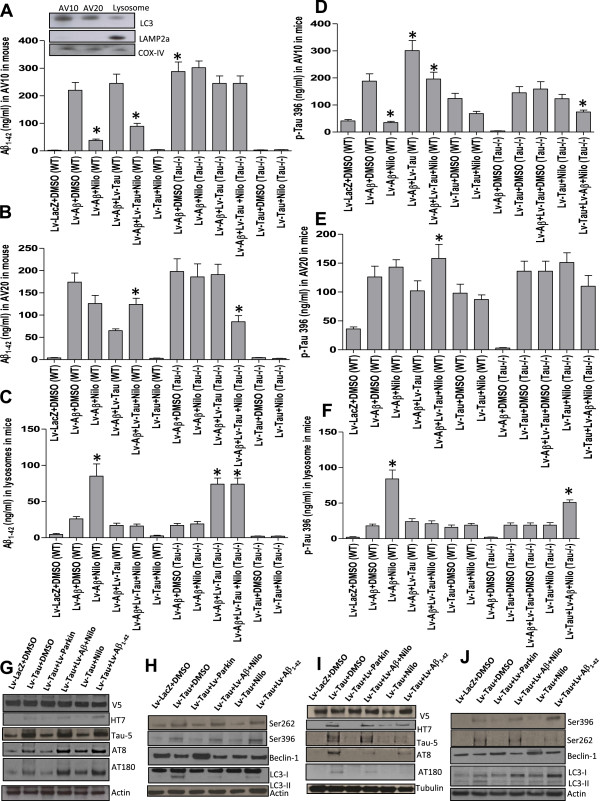
**Boosting autophagy leads to Aβ1-42 clearance in WT but not Tau** ^**−/−**^ **mice.** WT and Tau^−/−^ mice were injected with lentiviral Tau ± Aβ1-42 for 3 weeks and treated I.P. with 10 mg/kg Nilotinib or DMSO once a day for 3 weeks. Brain tissues were fractionated to isolate AVs and human specific ELISA was performed. Histograms represent concentration of **A)** Aβ1-42, insert is WB on 4-12% SDS NuPAGE gel showing LC3 and LAMP-2a as AV markers, **B)** Aβ1-42 in AV20, and **C)** Aβ1-42 in lysosomal fractions in WT and Tau^−/−^ mice. ELISA concentrations of Ser 396 Tau in **D)** AV10, **E)** AV20, and **F)** lysosomal fractions in WT and Tau^−/−^ mice. WB analysis on 4-12% SDS NuPAGE gel of total brain extracts from WT mice showing **G)** V5 to verify equal expression of all lentiviruses, human Tau (HT7), total Tau, AT8 and AT180 relative to actin. **H)** shows p-Tau Ser 262, Ser 396 and autophagic markers Beclin-1 and LC3-I/II relative to actin. WB analysis on 4-12% SDS NuPAGE gel of total brain extracts from Tau^−/−^ mice showing **I)** V5, human Tau (HT7), total Tau, AT8, and AT180 relative to tubulin. **J)** shows p-Tau Ser 262, Ser 396 and autophagic markers Beclin-1, LC3-I and LC3-II relative to actin. Asterisk indicates significantly different to Aβ1-42 + DMSO, bars are mean ± SEM, two-way ANOVA.

To determine autophagic flux, mouse brain tissues were fractionated to isolate autophagic vacuoles (AVs), which were identified by light chain protein-3 (LC3) that indicates pre-lysosomal autophagosome formation (Figure 3A, insert) as we previously indicated [14, 16]. Lysosome associated membrane protein (LAMP)-2a was used as a marker of the lysosomal fraction (Figure 3A, n = 4). Mitochondrial cytochrome c oxidase (COX)-IV was also used as another control marker. ELISA measurement of Aβ1-42 in AVs in WT mice expressing Aβ1-42 showed Aβ1-42 accumulation in AV10 (Figure 3A, n = 4) and AV20 (Figure 3B, n = 4) but not in the lysosome (Figure 3C, n = 4). Nilotinib significantly reduced Aβ1-42 in AV10 (Figure 3A, n = 4, p < 0.001) and increased it in the lysosome (Figure 3C, p < 0.0001), suggesting that Nilotinib facilitates autophagic flux from autophagosomal vacuoles to the lysosome [22]. In WT mice expressing Aβ1-42 and Tau together, Nilotinib significantly reduced Aβ1-42 in AV10 (Figure 3A, n = 4, p < 0.01) and increased it in AV20 (Figure 3B, p < 0.04). In Aβ1-42-expressing Tau^−/−^ mice, Aβ1-42 was significantly higher in AV10 (Figure 3A, n = 4, p < 0.03) and unchanged in AV20 (Figure 3B) or lysosomes (Figure 3C) compared to WT. Nilotinib did not alter Aβ1-42 levels in AVs in Tau^−/−^ mice, suggesting that Tau deletion affects flux through deposition of autophagosomal contents into the lysosomes. However, Nilotinib decreased Aβ1-42 in AV20 (Figure 3A, p < 0.05) and increased it in the lysosomes (Figure 3C, p < 0.001) when lentiviral Tau was co-expressed with Aβ1-42 in Tau^−/−^ mice, indicating that exogenous Tau restores Aβ1-42 clearance.

Further ELISA measurement of Ser396 p-Tau showed significantly high levels of p-Tau in AV10 (Figure 3D, n = 4, p < 0.0001) and AV20 (Figure 3E, p < 0.001) in Aβ1-42 expressing WT mice compared to LacZ (+DMSO), but Nilotinib reduced p-Tau in AV10 (Figure 3D, p < 0.001) and increased it in the lysosomes (Figure 3F, p < 0.001). Expression of Aβ1-42 together with Tau in WT mice significantly increased p-Tau in AV10 (Figure 3D, n = 4, p < 0.001) compared to Aβ1-42 alone, but Nilotinib again reduced p-Tau in AV10 (p < 0.03) and increased it in AV20 (Figure 3E, p < 0.03). Nilotinib also reduced p-Tau in AV10 (Figure 3D, p < 0.03) and AV20 (Figure 3E, p < 0.04) in Tau expressing WT mice. Lentiviral Tau expression led to detection of p-Tau in AV10 (Figure 3D) and AV20 (Figure 3E) but not in the lysosomes in Tau^−/−^ mice and Nilotinib did not alter p-Tau levels, suggesting that exogenous Tau may not affect autophagic p-Tau clearance. However, when Tau was co-expressed with Aβ1-42 in Tau^−/−^ mice, Nilotinib significantly decreased p-Tau in AV10 (Figure 3D, p < 0.01) and AV20 (Figure 3E, p < 0.05) and increased it in lysosomes (Figure 3F, p < 0.001), indicating that exogenous Tau affects Aβ1-42 and p-Tau clearance in Tau^−/−^ mice.

We previously demonstrated an effect for parkin and Nilotinib on autophagic Aβ1-42 clearance [14, 16], so we used lentiviral parkin expression as a control for Nilotinib to show markers of autophagic changes. WB of total brain lysates shows equal V5 levels (Figure 3G, n = 5) in WT mice, indicating equal expression of lentiviral clones. Human Tau (HT7) was only detected in WT mice expressing human Tau (Figure 3G) and was decreased in the presence of parkin or Nilotinib (n = 5, p < 0.05), in agreement with ELISA. However, total Tau (Tau-5) and p-Tau (AT8) were increased in Tau (n = 5, p < 0.05) and Tau and Aβ1-42 (p < 0.05) expressing WT mice, but both parkin and Nilotinib decreased p-Tau (38 and 50%, respectively, p < 0.04) relative to actin. Nilotinib significantly decreased p-Tau at Ser262 (44%) and Ser 396 (53%) relative to actin in Aβ1-42 alone or with Tau in WT mice (Figure 3H, n = 5, p < 0.05).

Tau expression decreased the autophagy enzyme, Beclin-1 compared to LacZ relative to actin (Figure 3H and Additional file 1: Figure S1 1 M, n = 5), but Nilotinib and parkin increased Beclin-1 levels (40% and 51%, respectively, p < 0.05) relative to actin when Aβ1-42 was expressed alone or together with Tau (Figure 3H and Additional file 1: Figure S1 1 M, n = 5). No significant changes in LC3-I levels (Figure 3H and Additional file 1: Figure S1 1 M, n = 5) were detected in WT mice but LC3-II was increased in Tau (100%) or Tau and Aβ1-42 (44%) relative to LC3-I or actin (Figure 3H and Additional file 1: Figure S1 1 M, n = 5, p < 0.001). LC3-II disappeared in Nilotinib and parkin mice, suggesting enhanced autophagosome clearance. Equal V5 levels (Figure 3I, n = 5) were also detected in Tau^−/−^ mice, and human Tau (HT7) was only detected in lentiviral Tau expressing Tau^−/−^ mice (Figure 3I) and disappeared in the presence of parkin or Nilotinib (n = 5). Total Tau (Tau-5) and p-Tau (AT8) were detected in Tau expressing mice but both parkin and Nilotinib cleared p-Tau relative to tubulin. Nilotinib eliminated Ser262 and Ser 396 p-Tau when Aβ1-42 was expressed alone or with Tau in Tau^−/−^ mice (Figure 3J, n = 5, p < 0.05). Tau expression decreased Beclin-1 compared to LacZ relative to actin (n = 5) but Nilotinib and parkin increased Beclin-1 (35% and 59%, respectively, p < 0.05) relative to actin levels when Aβ1-42 was expressed alone or together with Tau (Figure 3J and Additional file 1: Figure S1 1 M, n = 5). LC3-II significantly increased in Tau (58%) alone or with Aβ1-42 (48%) relative to LC3-I (n = 5, p < 0.05). LC3-II was reversed back to LacZ with Nilotinib and parkin, suggesting autophagosome clearance.

### Nilotinib reduces p-Tau when exogenous Tau is expressed in Tau^−/−^ mice

**Figure 4:**
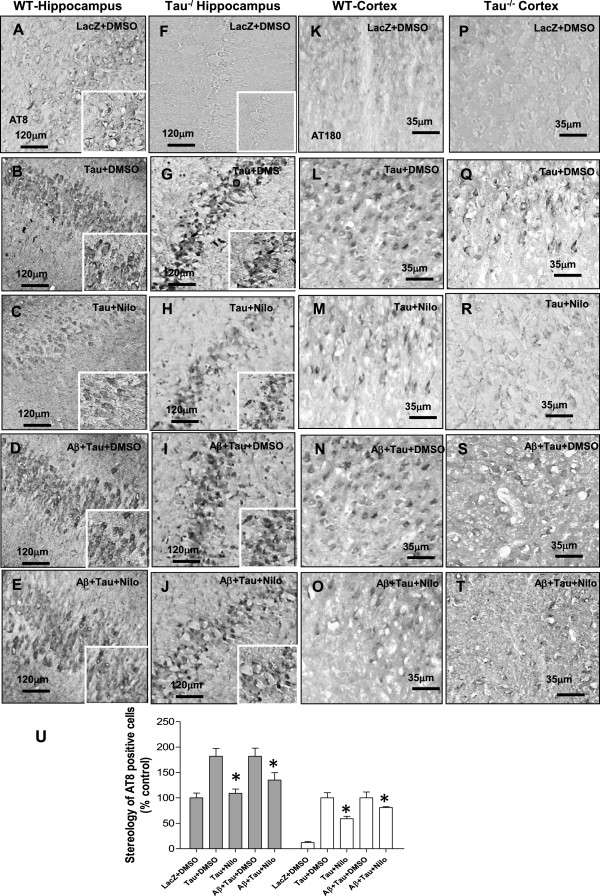
**Aβ1-42 is more efficiently cleared in WT than Tau** ^**−/−**^ **mice.** Staining of 20 μm thick coronal sections with p-Tau (AT8) and counterstained with DAB in **A)** LacZ + DMSO, **B)** Tau + DMSO, **C)** Tau + Nilotinib, **D)** Tau and Aβ1-42 + DMSO and **E)** Tau and Aβ1-42 + Nilotinib. Staining of hippocampus with AT8 and DAB in **F)** LacZ + DMSO, **G)** Tau + DMSO, **H)** Tau + Nilotinib, **I)** Tau and Aβ1-42 + DMSO and **J)** Tau and Aβ1-42 + Nilotinib in Tau^−/−^ mice. Staining of 20 μm thick coronal sections with p-Tau (AT180) and DAB in **K)** LacZ + DMSO, **L)** Tau + DMSO, **M)** Tau + Nilotinib, **N)** Tau and Aβ1-42 + DMSO and **O)** Tau and Aβ1-42 + Nilotinib in WT mice. Staining of cortical sections in **P)** LacZ + DMSO, **Q)** Tau + DMSO, **R)** Tau + Nilotinib, **S)** Tau and Aβ1-42 + DMSO and **T)** Tau and Aβ1-42 + Nilotinib in Tau^−/−^ mice. **U)** Histograms represent stereological quantification of p-Tau. Asterisk indicates significantly different to Aβ1-42 + DMSO, bars are mean ± SEM, two-way ANOVA.

We determined whether introduction of exogenous Tau affects p-Tau clearance in WT and Tau^−/−^ mice treated with Nilotinib. Labeling with AT8 and 3, 3’-diaminobenzidine (DAB) counterstaining shows endogenous p-Tau in the hippocampus of WT mice injected with a lentivirus driving LacZ expression (Figure 4A, n = 4, insert shows higher magnification). Human WT Tau increased (Figure 4U, 82%, by stereology, insert is higher magnification) p-Tau (Figure 4B, n = 5, p < 0.02) in WT mice treated with DMSO but Nilotinib (Figure 4C, insert is higher magnification) eliminated p-Tau (n = 5). Aβ1-42 and Tau together (Figure 4D, insert is higher magnification) increased p-Tau (Figure 4U, 82%, by stereology, p < 0.02) compared to LacZ but Nilotinib (Figure 4E) decreased p-Tau (Figure 4U, 35% higher than control, p < 0.04) in WT mice. No p-Tau was observed in the hippocampus of Tau^−/−^ mice (Figure 4F, insert is high magnification) but human Tau expression (Figure 4G, insert is higher magnification) led to p-Tau in Tau^−/−^ mice with DMSO, while Nilotinib reduced (Figure 4U, 41%, n = 5, p < 0.05) p-Tau (Figure 4H, insert is higher magnification). Co-expression of Aβ1-42 and Tau (Figure 4J, insert is higher magnification) increased p-Tau that was not different than Tau alone (Figure 4I) but Nilotinib reduced (19%) p-Tau (Figure 4J, n = 5, insert is higher magnification) compared to DMSO. Injection of lentiviral Tau into the hippocampus also increased p-Tau (AT180) (91%, by stereology) in the cortex in mice (Figure 4L, n = 5, p < 0.03) treated with DMSO compared to LacZ (Figure 4K), but Nilotinib (Figure 4M, n = 5) reduced p-Tau (45%, p < 0.05). Aβ1-42 and Tau together (Figure 4N, n = 5) increased p-Tau levels (69%, by stereology, p < 0.04) compared to LacZ but Nilotinib (Figure 4O) decreased p-Tau (62% by stereology, p < 0.04) in WT mice. No p-Tau was observed in the cortex of Tau^−/−^ mice (Figure 4P) but human Tau expression (Figure 4Q) increased p-Tau in Tau^−/−^ mice with DMSO, while Nilotinib reduced (56%, n = 5, p < 0.05) p-Tau (Figure 4R) compared to DMSO (Figure 4Q). Co-expression of Aβ1-42 and Tau increased p-Tau (Figure 4S, 29%) compared to Tau alone and Nilotinib significantly reduced p-Tau levels (Figure 4T, n = 5, 31%, p < 0.05) compared to DMSO (Figure 4U) in Tau^−/−^ mice.

### Tau deletion reduces intracellular Aβ1-42 and contributes to plaque formation

**Figure 5:**
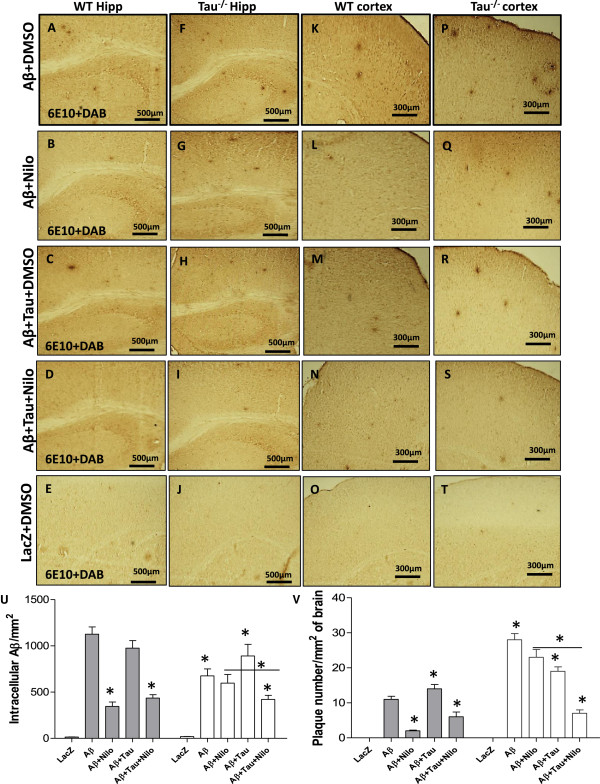
**Increased Aβ1-42 plaque deposition in Tau** ^**−/−**^ **mice.** Staining of 20 μm thick coronal sections with 6E10 and DAB in wild type mice injected with lentiviral **A)** Aβ1-42 + DMSO, **B)** Aβ1-42 + Nilo, **C)** Aβ1-42 + Tau + DMSO, **D)** Aβ1-42 + Tau + Nilo and **E)** LacZ + DMSO in the hippocampus. Staining of 20 μm thick coronal sections with 6E10 and DAB in Tau^−/−^ mice injected with lentiviral **F)** Aβ1-42 + DMSO, **G)** Aβ1-42 + Nilo, **H)** Aβ1-42 + Tau + DMSO, **I)** Aβ1-42 + Tau + Nilo and **J)** LacZ + DMSO in the cortex. Staining of 20 μm thick coronal sections with 6E10 and DAB in wild type mice injected with lentiviral **K)** Aβ1-42 + DMSO, **L)** Aβ1-42 + Nilo, **M)** Aβ1-42 + Tau + DMSO, **N)** Aβ1-42 + Tau + Nilo and **O)** LacZ + DMSO in the cortex. Staining of 20 μm thick coronal sections with 6E10 and DAB in Tau^−/−^ mice injected with lentiviral **P)** Aβ1-42 + DMSO, **Q)** Aβ1-42 + Nilo, **R)** Aβ1-42 + Tau + DMSO, **S)** Aβ1-42 + Tau + Nilo and **T)** LacZ + DMSO in the cortex. Histograms represent **U)** stereological counting of Aβ1-42 positive cells and **V)** plaque number/mm^2^ in total brain. Asterisk is significantly different to control (Aβ1-42 + DMSO) or as indicated, bars are mean ± SEM, two-way ANOVA.

We determined whether Tau deletion affects the distribution of intracellular and extracellular Aβ1-42. Plaque deposition was observed in the hippocampus of WT mice expressing Aβ1-42 at 1 month post-injection (Figure 5A, n = 5). Nilotinib significantly decreased (by stereology) intracellular Aβ1-42 (Figure 5B and U, n = 5, p < 0.01), which was also verified with human specific Aβ1-42 antibody staining as shown in Additional file 1: Figure S1. Nilotinib reduced plaque load (Figure 5B and V, p < 0.001) compared to DMSO (Figure 5A, n = 5) in WT mice expressing Aβ1-42 alone, consistent with our previously published data [14, 23]. Tau and Aβ1-42 increased plaque levels (Figure 5C and V, n = 5, p < 0.02) in WT mice treated with DMSO and Nilotinib significantly decreased intracellular Aβ1-42 (Figure 5D and U, p < 0.02) and plaque load (Figure 5D and V, p < 0.001) compared to Aβ1-42 alone or LacZ (Figure 5E and U, p < 0.001, n = 5). Nilotinib did not alter intracellular Aβ1-42 staining (Figure 5G and U, n = 5) or plaque load (Figure 5G and V) in the hippocampus of Tau^−/−^ mice compared to DMSO (Figure 5F, n = 5), which displayed more plaque (Figure 5F and V, p < 0.001, n = 5) and less intracellular Aβ1-42 (Figure 5F and U, p < 0.001) Aβ compared to WT mice (Figure 5A). Tau expression with Aβ1-42 in Tau^−/−^ mice increased intracellular Aβ1-42 (Figure 5H and U, p < 0.01, n = 5) and decreased plaque levels (Figure 5H and V, p < 0.05) while Nilotinib reversed intracellular Aβ1-42 (Figure 5I and U, p < 0.001) and plaque (Figure 5I and V, p < 0.001) back to WT levels (Figure 5D). No Aβ1-42 staining was observed in mice expressing LacZ (Figure 5J). Plaque deposition and intracellular Aβ1-42 were also detected in the cortex of WT mice expressing Aβ1-42 alone (Figure 5K) or together with Tau (Figure 5M) while Nilotinib reduced Aβ1-42 staining (Figure 5L and N) compared to DMSO (Figure 5K and I) and LacZ (Figure 5O, n = 5). Plaque deposition was higher in the cortex of Tau^−/−^ mice expressing Aβ1-42 alone (Figure 5P) or together with Tau (Figure 5R) while Nilotinib reduced Aβ1-42 staining only when Tau was introduced (Figure 5S) compared to Aβ1-42 alone (Figure 5Q and I) and LacZ (Figure 5T, n = 5).

### Tau deletion attenuates Aβ1-42-induced cell death despite the increase in plaque load

**Figure 6:**
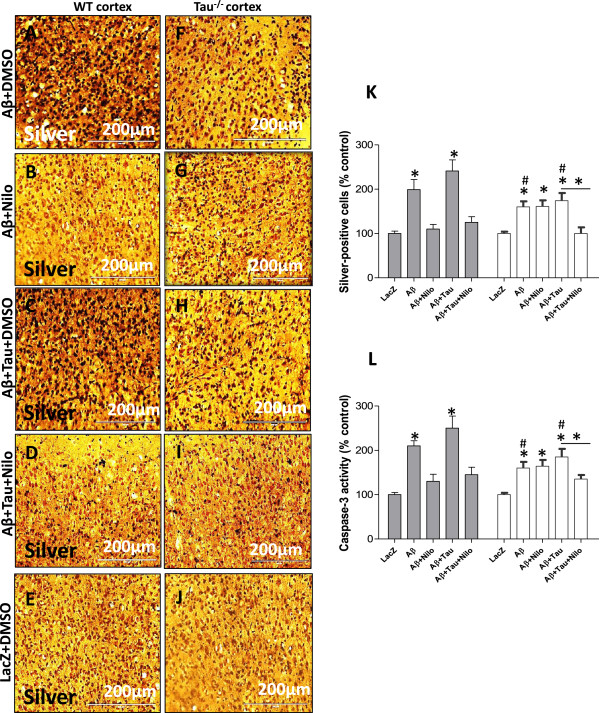
**Tau deletion attenuates Aβ1-42-induced cell death.** Cupric silver staining of 20 μm thick coronal sections in WT mice injected with lentiviral **A)** Aβ1-42 + DMSO, **B)** Aβ1-42 + Nilo, **C)** Aβ1-42 + Tau + DMSO, **D)** Aβ1-42 + Tau + Nilo and **E)** LacZ + DMSO. Silver staining in Tau^−/−^ mice injected with lentiviral **F)** Aβ1-42 + DMSO, **G)** Aβ1-42 + Nilo, **H)** Aβ1-42 + Tau + DMSO, **I)** Aβ1-42 + Tau + Nilo and **J)** LacZ + DMSO. Histograms represent **K)** stereological quantification of silver-positive cells and **L)** caspase-3 activity. Asterisk is significantly different to Aβ1-42 + DMSO or as indicated, bars are mean ± SEM, two-way ANOVA.

To determine whether Tau deletion affects cell viability in parallel with the distribution of intracellular and plaque Aβ1-42, cell death was assessed via silver staining that detects degenerating fibers and neurons and caspase-3 activity. Aβ1-42 expression (+DMSO) increased the number of silver-positive cells (Figure 6A and K, n = 5, p < 0.05) compared to LacZ (Figure 6E and K) in WT mice and Nilotinib eliminated cell death (Figure 6B). An increase in silver-stained cells was detected when Tau was expressed together with Aβ1-42 (Figure 6C and K, n = 5, p < 0.05) and again Nilotinib reduced cell death (Figure 6D and K) in WT mice. In Tau^−/−^ mice, Aβ1-42 (+DMSO) also increased the number of silver-positive cells (Figure 6F and K, n = 5, p < 0.05) compared to LacZ (Figure 6J and K) but this increase remained significantly lower than WT (Figure 6A and K, p < 0.05), suggesting that Tau deletion attenuates Aβ1-42 toxicity. In contrast with WT mice, Nilotinib did not reduce Aβ1-42-induced cell death in Tau^−/−^ mice (Figure 6G). Exogenous Tau and Aβ1-42 together increased cell death (Figure 6H and K, n = 5, p < 0.01), which remained lower than WT (Figure 6A) but Nilotinib completely reversed cell death (Figure 6I and K), indicating that Tau is needed to mediate autophagic clearance and reduce Aβ1-42 toxicity. Caspase-3 activity was also increased in WT mice expressing Aβ1-42 alone or together with Tau (Figure 6L, n = 5, p < 0.025) but Nilotinib reversed these effects. Although Aβ1-42 increased caspase-3 activity with and without Tau (Figure 6L, n = 5, p < 0.05), Nilotinib reversed these effects only when Tau was co-expressed with Aβ1-42.

## Discussion

These studies demonstrate that autophagic intracellular Aβ1-42 clearance requires Tau, suggesting that normal Tau function modulates plaque deposition via regulation of intracellular Aβ1-42 degradation. Inhibition of either the proteasome or autophagy led to partial Aβ1-42 and p-Tau clearance, suggesting that Aβ1-42 and p-Tau may be degraded via either autophagy and/or the proteasome. Tau deletion impaired intracellular Aβ clearance and increased extracellular plaque formation, while introduction of human Tau into Tau^−/−^ brains restored autophagic Aβ1-42 and p-Tau clearance and reduced plaques. We previously demonstrated that the E3 ubiquitin ligase parkin is essential for Nilotinib-induced autophagic amyloid clearance [14]. However, parkin and Tau differentially alter autophagic flux. Parkin deletion affects the transfer of Aβ1-42 and p-Tau from pre-lysosomal AVs, suggesting impairment of the earlier steps of the sequestration process [14, 16]. Tau deletion affects the deposition of amyloids from AVs into the lysosomes, indicating that Tau is required for the completion of autophagic clearance. However, impairment of autophagic flux with either Tau or parkin deletion leads to plaque deposition, further suggesting that reduction of intracellular Aβ1-42 clearance may lead to its secretion. Nilsson et al. [24] recently showed that blocking autophagy via ATG7 deletion in AD mouse diminishes extracellular Aβ release, increases its intracellular level and reduces extracellular amyloid plaque. Our results suggest that Tau deletion reduces intracellular Aβ level and increases plaque build-up. Nilsson et al. [24] model inhibited autophagy at an early pre-autophagosomal level where an ubiquitin-like reaction involving ATGs triggers autophagy, leading to autophagosome formation and subsequent maturation [22]. Our model demonstrated impairment of autophagic flux at a later pre-lysosomal stage, where Aβ and/or Tau have already accumulated in autophagosomes without deposition into the lysosomes. Thus, Aβ release and plaque formation may depend on the type of impairment along the autophagic cascade, suggesting that pre-autophagosomal inhibition of flux results in intracellular accumulation and less Aβ secretion whereas arrest of autophagic flux after Aβ deposition in undigested autophagosomes leads to Aβ secretion and plaque formation.

Tau maybe required to facilitate autophagosome maturation and retrograde transport from distal axons towards cell bodies to fuse with lysosomes [25], thus contributing to modulation of autophagic flux. Our data suggest that Tau deletion reduces intracellular Aβ degradation [14], which may be due in part to axonal changes that affect efficient autophagosome fusion with the lysosome in the Tau^−/−^ mouse, which is reported to have impaired organelle movement [20, 21]. The normal role of Tau in autophagy was shown in cellular models of Niemann-Pick type C (NPC) disease showing that acute reductions of Tau in NPC1-deficient fibroblasts impairs autophagy [26]. As autophagy is up-regulated in NPC and protein degradation may depend on movement along microtubules, Tau knockdown aggravates NPC pathology through a mechanism independent of Tau aggregation [27] as is often observed in neurodegenerative diseases, further suggesting a critical role for normal Tau in the regulation of autophagy. Taken together these data raise the possibility that loss of Tau function via mutations, deletion or hyper-phosphorylation may lead to decreased Tau binding to microtubules and subsequent impairment of autophagic flux, which may alter the distribution of intracellular and extracellular Aβ1-42 overtime. Tau may affect autophagic flux via interaction with autophagy enzymes, including Beclin-1 and the microtubule associated protein A/B light chain (MAPA/BLC)-3. We previously showed that p-Tau co-localizes with LC3 in rat brains expressing WT or mutant P301L Tau [28] and the elucidation of the molecular mechanisms underlying interaction between Tau and autophagy proteins is an area for future investigation.

A number of studies suggested that facilitation of Tau clearance via up-regulation of the proteasome or autophagy can protect cells from age-related stress [29, 30]. Previous studies showed that impaired autophagy may affect proteasomal activity [31], but the interplay between autophagy and the proteasome may not be a simple compensatory relationship. Our studies show that loss of normal Tau function via deletion impairs autophagic flux, independent of proteasomal activity. We demonstrated that introduction of WT Tau into Tau^−/−^ mice partially restores autophagic Aβ1-42 clearance, indicating that exogenous human Tau expression may affect Aβ1-42 levels. Expression of exogenous Tau may restore Aβ1-42 clearance due to possible integration of Tau into microtubules, leading to facilitation of autophagy. However, the relationship between Aβ and Tau is contentious with more recent *in vivo* reports demonstrating that Tau deletion prevents Aβ toxicity, and previous cell culture studies showing that Aβ triggers Tau pathology (reviewed in [32]). Our data show that Tau deletion exacerbates extracellular plaque build-up but attenuates cell death, indicating that Tau may modulate Aβ1-42 toxicity via alteration of intracellular and extracellular Aβ1-42 levels. Clinically, some aged human brains have plaques with no associated dementia or major cognitive decline [10, 11]. However, Aβ and Tau together are often associated with dementia [12] and Tau mutations and/or accumulation are causal to some neurodegenerative diseases that do not display β-amyloid pathology, including FTDP-17, PSP, and CBD, suggesting that Tau associated neurodegeneration can occur independently of plaque deposition [6, 7]. Taken together, these clinical findings support our hypothesis that Tau may be a critical modulator of Aβ1-42 toxicity through autophagic clearance of toxic intracellular Aβ1-42 [13], which may be secreted when autophagy fails, leading to extracellular plaque deposits.

In conclusion, loss of normal Tau function may alter microtubule stability and affect the execution of autophagy. Our findings agree with previous studies that basal autophagy is essential and elevated autophagic activity is beneficial for neurons to prevent accumulation of protein aggregates [33–36]. The current studies demosntrate that Tau is required to mediate the beneficial effects of autophagic clearance. Boosting autophagy was shown to reduce free unbound p-Tau and spare microtubule associated protein Tau in models of neurodegeneration [14, 37]. The current studies suggest that Tau function regulates intracellular Aβ1-42 clearance and affects extracellular plaque deposition.

## Methods

### Primary hippocampal neuronal culture

Fetal WT hippocampal tissue (gestation E15) were dissected and gently triturated with a fire-polished Pasteur pipette, and the resulting pool of dissociated cells were plated in 35-mm dishes precoated with polyethyleneimine (1 mg/ml) (MP Biomedicals NC-N19544450) containing 0.5 ml basal growth medium, which was supplemented with 5% horse serum and 0.5% fetal calf serum and replaced with fresh medium every three days. DIV 14 or when the density of cells reached 3×10^4^ cells, neurons were infected with 3 μl lentiviruses for 24 hrs and then treated with 10 μM Nilotinib (AMN-107, NC0604306, SELLECK CHEMICAL LLC) or 1 μL DMSO for 24 hrs or 100 nM Bafilomycin-A1(AC32812-0001, Acros Organics) or 20 μM MG132 (NC9819784, Cayman Chemicals) for 6 hrs.

### Stereotaxic injection

Lentiviral constructs driving LacZ, human WT Tau and/or Aβ1-42 were stereotaxically injected at 1×10^6^ multiplicity of infection (MOI) into the right CA1 hippocampus of 1 year old male C57BL/6 or Tau^−/−^ as we previously explained [14, 18]. All procedures were approved by the Georgetown University Animal Care and Use Committee (GUACUC). Nilotinib was dissolved in DMSO and a total volume of 30 μl was I.P. injected once a day for 3 weeks. Half the animals received DMSO and the other half received Nilotinib in DMSO.

#### WB analysis

To extract the soluble protein fraction, brain tissue or cells were isolated and homogenized in 1× STEN buffer (50 mM Tris (pH 7.6), 150 mM NaCl, 2 mM EDTA, 0.2% NP-40, 0.2% BSA, 20 mM PMSF and protease cocktail inhibitor), centrifuged at 10,000 g for 20 min at 4°C and the supernatant containing the soluble protein fraction was collected. To extract insoluble Aβ1-42, the pellet was re-suspended in 30% formic acid and centrifuged at 10,000 g for 20 min at 4°C and the supernatant was collected. Extracts were analyzed by WB on SDS NuPAGE 4-12% Bis-Tris gel (Invitrogen, NP0301BOX). β-actin was probed (1:1000) with polyclonal antibody (ThermoScientific, PA121167). Autophagy antibodies, including Beclin-1 (1:1000) and LC3-B (1:1000) were used to probe according to autophagy antibody sampler kit (Cell Signaling, Inc. Cat# 4445). Tau antibodies were used as we previously described [14, 18]. WBs were quantified by densitometry using Quantity One 4.6.3 software (Bio Rad).

#### IHC of brain sections

Animals were deeply anesthetized with a mixture of (1:8) Xylazine (Edgewood Pharmacy, 20130822) and Ketamine (Butler Animal Health Supply, 023061), washed with 1× saline for 1 min and then perfused with 4% (PFA) paraformaldehyde (MP Biomedicals, ICN15014601) for 15–20 min. Brains were quickly dissected out and immediately stored in 4% PFA for 24 hrs at 4°C and then transferred to 30% sucrose at 4°C for 48 h. Tissues were cut using a cryostat at 4°C into 20 μm thick sections and stored at −20°C. Cupric silver and immuno-staining were performed as we previously described [14, 18].

*Stereological methods* were applied by a blinded investigator using unbiased stereology analysis (Stereologer, Systems Planning and Analysis, Chester, MD) to determine the total positive cell counts in 20 cortical/hippocampal fields on at least 10 brain sections (~400 positive cells per animal) from each animal.

Quantification of plaque load or counting plaque number was performed by a blind investigator using ImageJ by drawing a line around individual plaques within 1 mm^2^ radius of 6 randomly selected hippocampal and cortical regions in 6E10 stained slides. The number of plaques was averaged per mm^2^ and compared between treatment condition, as we previously described [14, 18].

### Subcellular fractionation for isolation of autophagic compartments

A total of 0.5 g of animal brains were homogenized at low speed (Cole-Palmer homogenizer, LabGen 7, 115 Vac) in 1× STEN buffer and centrifuged at 1,000 g for 10 min to isolate the supernatant from the pellet. The pellet was re-suspended in 1× STEN buffer and centrifuged once to increase the recovery of lysosomes. The pooled supernatants were then centrifuged at 100,000 rpm for 1 hr at 4°C to extract the pellet containing autophagic vacuoles (AVs) and lysosomes. The pellet was re-suspended in 10 ml (0 .33 g/ml) 50% Metrizamide (Acros Organics, AC22943-0250) and 10 ml in cellulose nitrate tubes. A discontinuous Metrizamide gradient was constructed in layers from bottom to top as follows: 6 ml of pellet suspension, 10 ml of 26%; 5 ml of 24%; 5 ml of 20%; and 5 ml of 10% Metrizamide [38]. After centrifugation at 10,000 rpm for 1 hr at 4°C, the fraction floating on the 10% layer (Lysosome) and the fractions banding at the 24%/20% (AV 20) and the 20%/10% (AV10) Metrizamide inter-phases were collected by a syringe and examined.

### 20S proteasome activity assay

Human M17 neuroblastoma cells were co-transfected with 3 μg Tau, LacZ or Aβ1-42 cDNAs for 24 hrs and then treated with 10 μM Nilotinib for additional 24 hrs (48 hrs total). Either 20 μM proteasomal inhibitor MG132 or 100nM autophagy inhibitor Bafilomycin-A1 were applied for 6 hrs prior to the beginning of proteasome activity assays. Cell extracts (100 μg) were incubated with 250 μM of the fluorescent 20S proteasome specific substrate Succinyl-LLVY-AMC (Enzo Life Sciences, BML-P802-0005) at 37°C for 2 hrs. The medium was discarded and proteasome activity was measured in tissue homogenates

### Aβ and p-Tau enzyme-linked immunosorbent assay (ELISA)

Specific p-Tau ser396 (Invitrogen, KHB7031) and Aβ_1–42_ (Invitrogen, KHB3442,) ELISA were performed according to manufacturer’s protocol as described [15, 19]. Caspase-3 activity assays were performed according to manufacturer’s protocol as we previously described [14, 18].

### Statistical analysis

Data were analyzed with GraphPad software (GraphPad Prism, CA) using two-way ANOVA. The number of experiments and p values are indicated in the text.

qRT-PCR in primary hippocampal neuronal culture was performed on Real-time OCR system with Fast SYBR-Green PCR master Mix (Applied Biosystems, NC0381818) in triplicate from reverse-transcribed cDNA from mouse primary cortical neurons injected with lentiviral LacZ, Tau and/or Aβ1-42 (24 hrs) treated with DMSO or Nilotinib (24 hrs) using the same primers that were utilized to clone human Aβ1-42 [13] or Tau [19] into the lentivirus. Gene expression values were normalized using GADPH levels.

## Electronic supplementary material

Additional file 1: Figure S1: To verify equal expression of lentiviral clones, 20 μm thick coronal brain sections were stained human specific **A)** Aβ1-42 and **B)** V5 and **C)** merged figure showing that both LacZ and Aβ1-42 were co-expressed. Endogenous phosphorylated Tau using **D)** AT8 and **E)** V5 and **I)** merged figure showing LacZ expression. Lentiviral epitope **G)** V5 and **H)** AT8 and **I)** merged figure showing that both LacZ and Tau were co-expressed. Human specific **J)** Aβ1-42, **K)** AT8 and **L)** merged figure shows that Tau and of Aβ1-42 were co-expressed. Histograms represent **M)** densitometry of Beclin-1 relative to actin and LC3-II relative to LC3-I in WT and Tau^−/−^ mice. Asterisk is significantly different to Aβ1-42 + DMSO, bars are mean ± SEM, two-way ANOVA. (TIFF 11 MB)
