# Supplementary figures and images for: Tau deletion impairs intracellular β-amyloid-42 clearance and leads to more extracellular plaque deposition in gene transfer models
Source: Mol Neurodegener. 2014 Nov 10;9:46. doi: 10.1186/1750-1326-9-46 (PMC4247762; doi:10.1186/1750-1326-9-46)

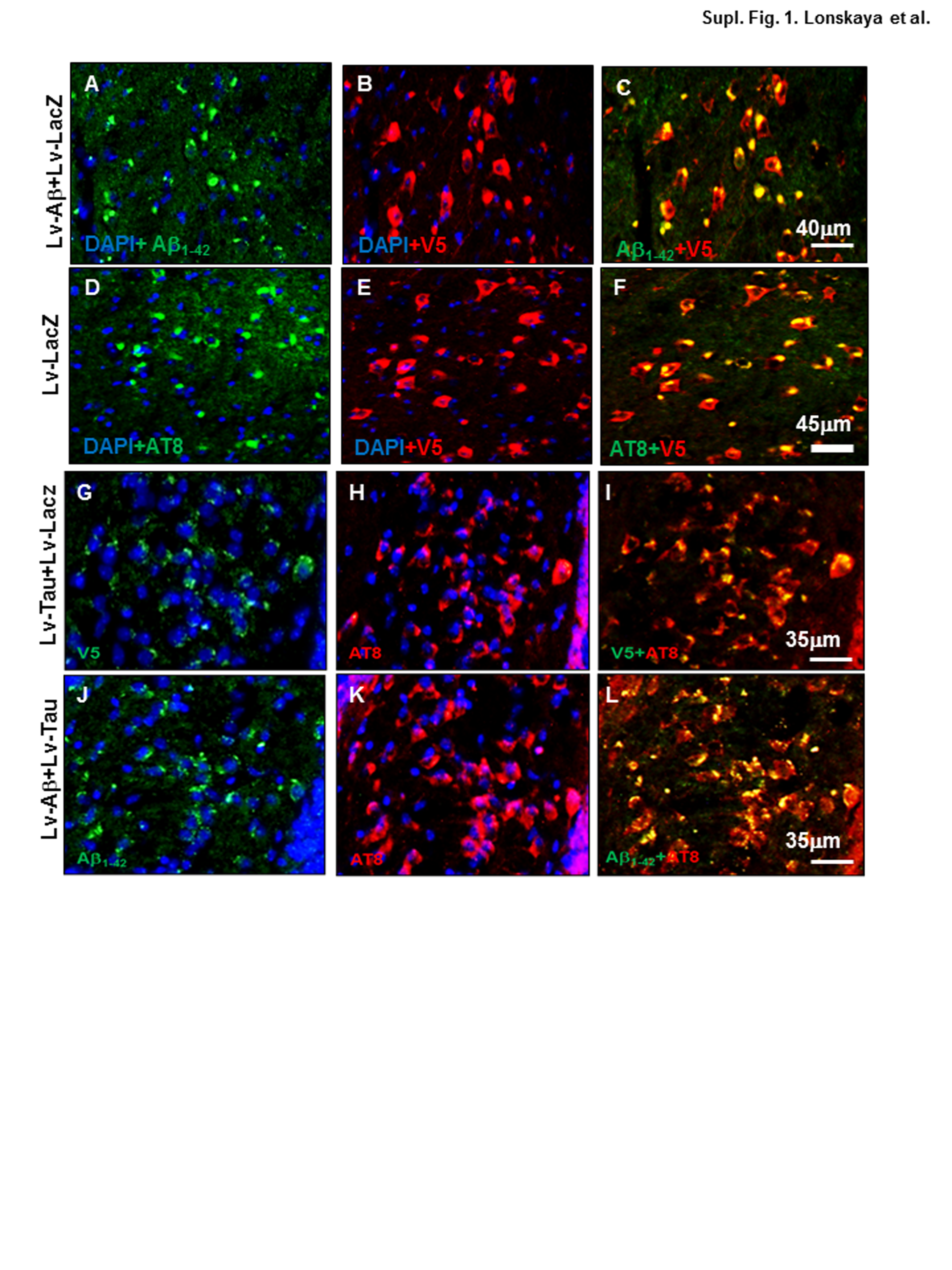

Supplement: Supplementary file 1 — Additional file 1: Figure S1: To verify equal expression of lentiviral clones, 20 μm thick coronal brain sections were stained human specific A) Aβ1-42 and B) V5 and C) merged figure showing that both LacZ and Aβ1-42 were co-expressed. Endogenous phosphorylated Tau using D) AT8 and E) V5 and I) merged figure showing LacZ expression. Lentiviral epitope G) V5 and H) AT8 and I) merged figure showing that both LacZ and Tau were co-expressed. Human specific J) Aβ1-42, K) AT8 and L) merged figure shows that Tau and of Aβ1-42 were co-expressed. Histograms represent M) densitometry of Beclin-1 relative to actin and LC3-II relative to LC3-I in WT and Tau−/− mice. Asterisk is significantly different to Aβ1-42 + DMSO, bars are mean ± SEM, two-way ANOVA. (TIFF 11 MB) [file 13024_2014_560_MOESM1_ESM.tiff]
